# Clinical characteristics, cerebellar MR spectroscopy and response to 3,4-diaminopyridine in spinocerebellar ataxia 27B: the Sheffield Ataxia Centre experience

**DOI:** 10.1007/s00415-025-13422-4

**Published:** 2025-10-09

**Authors:** Ikechukwu Chukwuocha, David Pellerin, Priya Shanmugarajah, Theocharis Tsironis, Emma Foster, Nigel Hoggard, Nick Beauchamp, Lauren Turton, Alisdair McNeill, Bernard Brais, Marios Hadjivassiliou

**Affiliations:** 1 Academic Department of Neurosciences, Sheffield Teaching Hospitals NHS Trust and University of Sheffield, Sheffield, UK; 2 Department of Neurology and Neurosurgery, Montreal Neurological Hospital and Institute, McGill University, Montreal, QC Canada; 3 Department of Neuromuscular Disease, UCL Queen Square Institute of Neurology and The National Hospital for Neurology and Neurosurgery, University College London, London, UK; 4 Department of Infection, Immunity and Cardiovascular Disease, University of Sheffield, Sheffield, UK; 5 Sheffield Diagnostic Genetics Service, Sheffield Children’s NHS Trust, Sheffield, UK; 6 Division of Neuroscience & Neuroscience Institute, The University of Sheffield, Sheffield, UK; 7 Sheffield Clinical Genetics Department, Sheffield Children’s Hospital NHS Foundation Trust, Sheffield, UK; 8 Department of Human Genetics, McGill University, Montreal, QC Canada; 9 Academic Department of Neurosciences, Royal Hallamshire Hospital, 16 Claremont Crescent, Room 12, Glossop Road, Sheffield, S10 2JF UK

**Keywords:** FGF14, GAA-FGF14 ataxia, Cerebellar ataxia, Genetics, MR spectroscopy, 3,4-Aminopyridine

## Abstract

**Background:**

The clinical and genetic heterogeneity of hereditary ataxias presents a significant diagnostic challenge, particularly in sporadic adult-onset cases. Spinocerebellar ataxia type 27B (SCA27B) is caused by an intronic GAA·TTC repeat expansion in the fibroblast growth factor 14 (F*GF14*) gene and is inherited in an autosomal dominant manner, although with reduced penetrance. This novel ataxia is emerging as a frequent yet underdiagnosed cause of late-onset often sporadic cerebellar ataxia.

**Method:**

In this study, we describe our experience in clinical presentation, neuroimaging characteristics (including MR spectroscopy of the cerebellum), tremor analysis, and therapeutic response to 3,4-diaminopyridine in a cohort of 50 patients with SCA27B.

**Results:**

The mean age at onset was 61.8 years. Episodic symptoms were reported in 28% of cases, while downbeat nystagmus and oscillopsia were observed in 50% and 28% individuals, respectively. Tremor was also present in 22% of patients. Tremor analysis demonstrated bilateral, intermediate-frequency (~ 6 Hz) tremor with both action and resting components, occasionally involving, apart from arms, the lower limbs and head. MRI findings revealed involvement of the superior cerebellar peduncle, and MR spectroscopy of the cerebellum (MRS) demonstrated a progressive decline in NAA/Cr area ratios in the cerebellar hemisphere over time. Notably, treatment with 3,4-diaminopyridine was associated with subjective symptom improvement in most patients and objective stabilization and/or improvement on MRS.

**Conclusion:**

Our findings expand the clinical, neuroimaging and tremor phenotype of SCA27B and support the use of 3,4-diaminopyridine as a potentially effective therapy.

## Introduction

Hereditary ataxias encompass a wide range of neurological disorders characterized primarily by progressive cerebellar ataxia. The list of causative genes is extensive and continues to expand, with several new genes discovered over the last few years [1]. Among the spinocerebellar ataxia syndromes, spinocerebellar ataxia 27B (SCA27B), an autosomal dominant genetic disorder caused by GAA·TTC repeat expansion in the first intron of the *FGF14* gene, which encodes the fibroblast growth factor 14, is one of the most prevalent causes of late-onset ataxia [2]. It accounts for between 9 and 61% of previously undiagnosed cases in different ethnically diverse ataxia cohorts [1, 3].

SCA27B is characterized by progressive cerebellar syndrome with more impact on gait, posture, and lower limbs than on upper extremities [4]. Like other forms of SCAs, individuals with this ataxia may manifest phenotypic heterogeneity, typified by impaired coordination and imbalance resulting in ataxia of stance and gait, limb ataxia, dysarthria, and, in particular, oculomotor signs (downbeat nystagmus-DBN) often resulting in oscillopsia. Less well characterized features include tremor and peripheral neuropathy [5]. The clinical progression tends to be slower than other late-onset ataxias and symptoms are often episodic, at least at the onset [4]. More importantly, some of these features respond to medical therapy with aminopyridine compounds [4, 6].

At the Sheffield Ataxia Centre, we care for over 3000 patients with progressive ataxia. Once genetic testing for SCA27B became possible, we were able to test a cohort of patients with an as yet unknown cause of their ataxia. We envisage that this may prove to be one of the commonest SCAs, at least in the UK. As such, our study investigated the clinical features, neuroimaging findings (including MRI and cerebellar MRI spectroscopy), tremor characteristics, and treatment response to 3, 4 DAP in this cohort with SCA27B ataxia to address the knowledge gap in the diagnosis and treatment of this condition.

## Methods

This retrospective, single-centre case series included 50 patients diagnosed with SCA27B presenting with late-onset (starting after the age of 30) [7] progressive cerebellar ataxia with or without a family history. The presence of downbeat or prominent nystagmus was one of the selection criteria we used for testing for this novel ataxia. All participants underwent genetic testing to assess for FGF14 GAA·TTC repeat expansions following informed consent for genetic testing. The diagnosis of GAA-FGF14–related ataxia was confirmed by identifying an intron 1 FGF14 (GAA) repeat expansion of > 300 through genetic testing. For repeat sizes of 250–300, diagnosis was established when the clinical presentation was consistent, alternative genetic causes had been excluded, and, where possible, familial segregation was demonstrated [8]. Molecular genotyping of the *FGF14* repeat locus was performed using capillary electrophoresis of fluorescent long-range PCR amplification product [9]. Pathogenic expansions were defined as ≥ 250 GAA·TTC repeat units [3, 10], confirming the molecular diagnosis of SCA27B. Additionally, as part of the series we identified eight individuals with GAA·TTC expansion < 250 not meeting the pathogenic threshold.

Clinical data were extracted from medical records using a standardized, Excel-based data collection tool. Collected variables included demographic information (age at symptom onset, age at evaluation or death, estimated disease duration) and a family history of ataxia.

Phenotypic characterization encompassed the distribution of ataxia (axial, appendicular, or mixed), associated oculomotor abnormalities (including presence and direction of nystagmus and oscillopsia), dysarthria, tremor, sensory, and episodic symptoms.

Neuroimaging findings were reviewed when available, including brain MRI (with a focus on cerebellar structures such as the superior cerebellar peduncle) and* a* single-voxel MR spectroscopy to evaluate cerebral neuronal integrity (N-acetyl-aspartate), measuring regional metabolite concentrations. N-acetyl-aspartate to creatine (NAA/Cr) ratios were obtained from the vermis and right cerebellar hemisphere and served as markers of cerebellar integrity. We have previously demonstrated good correlation between MR spectroscopy and the SARA score and the usefulness of this measurement as a monitoring tool [11].

Detailed neurophysiological analysis, including EEG and polygraphic surface EMG recordings as well as jerk-locked back averaging was performed in 4 patients with more complex tremor phenotypes to try and better characterize tremor frequency, distribution, and type.

Any response to 3,4-diaminopyridine (3,4-DAP) was documented and was assessed qualitatively based on patient-reported improvement in symptom frequency or severity. Missing or incomplete data were flagged during data processing and excluded from specific analyses as appropriate. Descriptive statistical analyses, including calculations of mean and median age at onset, and charts were plotted to summarize the cohort's demographic and clinical characteristics.

## Results

**Fig. 1:**
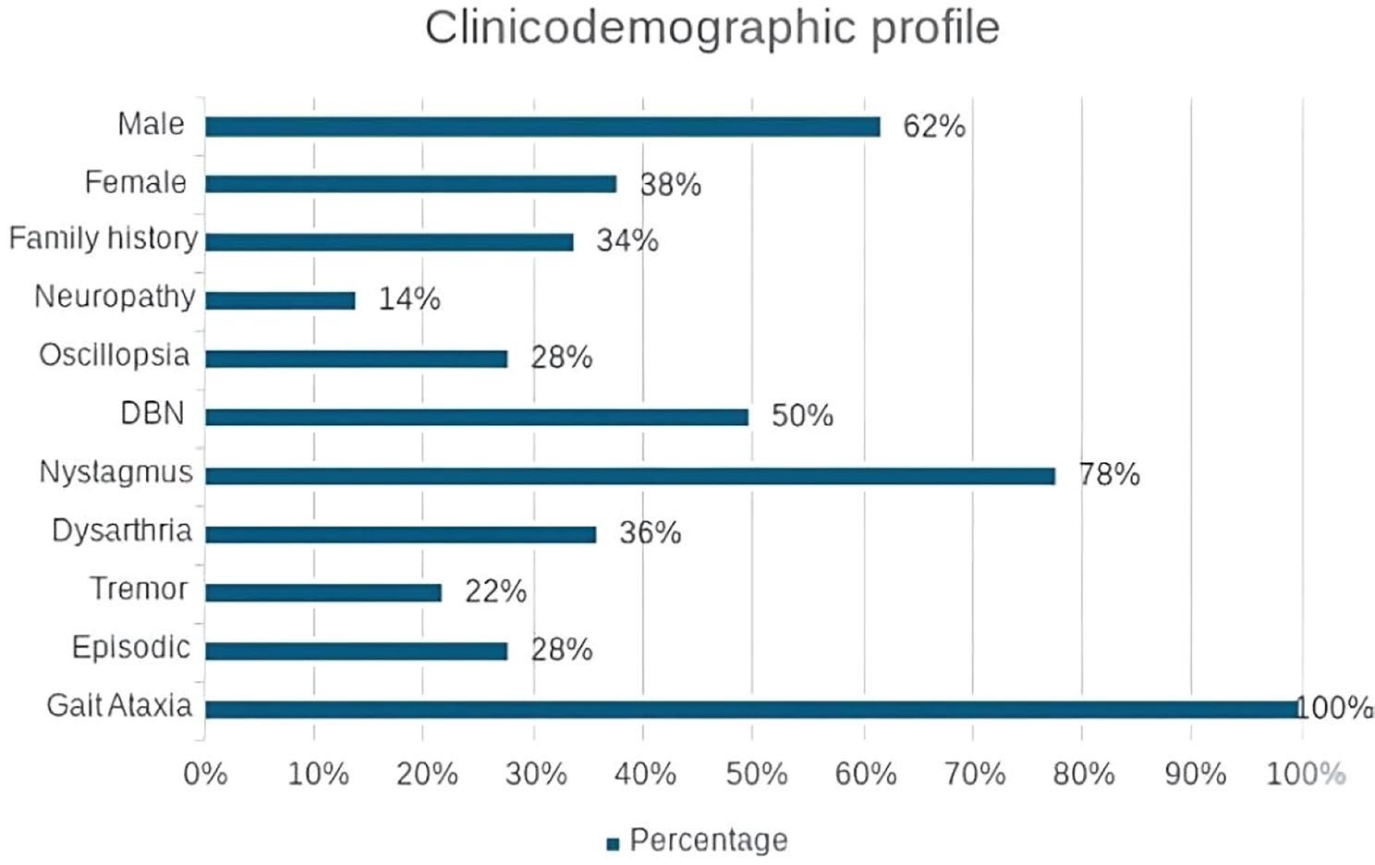
Demographics and Clinical Profile of 50 SCA27B Patients. DBN: downbeat nystagmus

This was, to our knowledge, the largest UK cohort of SCA27B cases from a single centre, with a total of 50 patients. The demographics and clinical characteristics of the case cohort (*n* = 50) are depicted in Fig. 1. The data revealed a male predominance (65.22%) and a mean age of symptom onset of 61.8 ± 14.3 (25–85). At the presentation, all patients reported gait unsteadiness. DBN was observed in 50% of patients, and gaze-evoked horizontal nystagmus was noted in the remaining 28%. Twenty-eight percent of our cohort reported episodic symptoms, particularly visual disturbances (oscillopsia, blurred vision and double vision) and gait unsteadiness. The episodic symptoms lasted from minutes to hours. Other accompanying neurological symptoms are shown in Fig. 1. While not all patients underwent electrophysiological assessment for the diagnosis of neuropathy, testing was performed in ten individuals who had symptoms and signs suggestive of neuropathy, revealing evidence of neuropathy in seven. Among these, four were identified as having sensorimotor axonal length-dependent neuropathy, while three demonstrated features consistent with sensory ganglionopathy.

Among the cohort, 11 (22%) exhibited tremor as a prominent feature. Neurophysiological tremor analysis was conducted in four patients, revealing a bilateral, intermediate-frequency (~ 6 Hz) tremor with both action and resting components, occasionally involving the lower limbs and head. Among the remaining seven patients not assessed by electrophysiology, various tremor types were reported on clinical assessment, including combined action/rest tremor (*n* = 2), intention tremor (*n* = 2), orthostatic tremor (*n* = 1), jerky action tremor (*n* = 1), and facial tremor/myokymia (*n* = 1). One patient also exhibited exaggerated startle, with no other hyperkinetic movements observed.

Disability varied from mild (*n* = 12), moderate (*n* = 20), and severe (*n* = 13). Up-to-date data were not available in 5 patients. Mild symptoms refer to individuals who can walk independently, while moderate and severe symptoms indicate those who require assistance (a walking stick or other aid for moderate cases) and a wheelchair for severe cases.

**Fig. 2:**
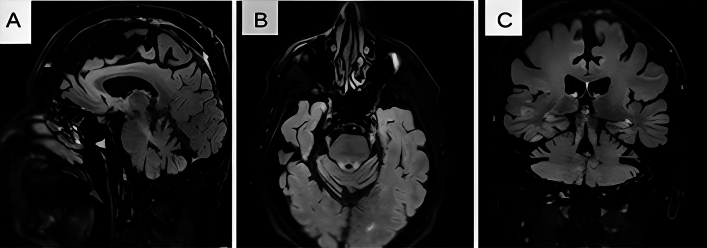
T2 FLAIR magnetic resonance imaging of the brain **A** sagittal view, **B** axial, and **C** coronal views, showing high signal intensity in the superior cerebellar peduncle and atrophy of the superior cerebellar vermis

A qualitative assessment of the patient's MR images revealed involvement of the superior cerebellar peduncle with prominent hyperintensity on T2-weighted sequences in the majority of cases (75.5%) out of the available 41 MRI images, and predominant atrophy of the cerebellar vermis. Additionally, supratentorial cerebral atrophy was observed in some individuals but this could simply be age related. A typical MRI brain of one of the patients is shown in Fig. 1 (see Fig. 2).

**Fig. 3:**
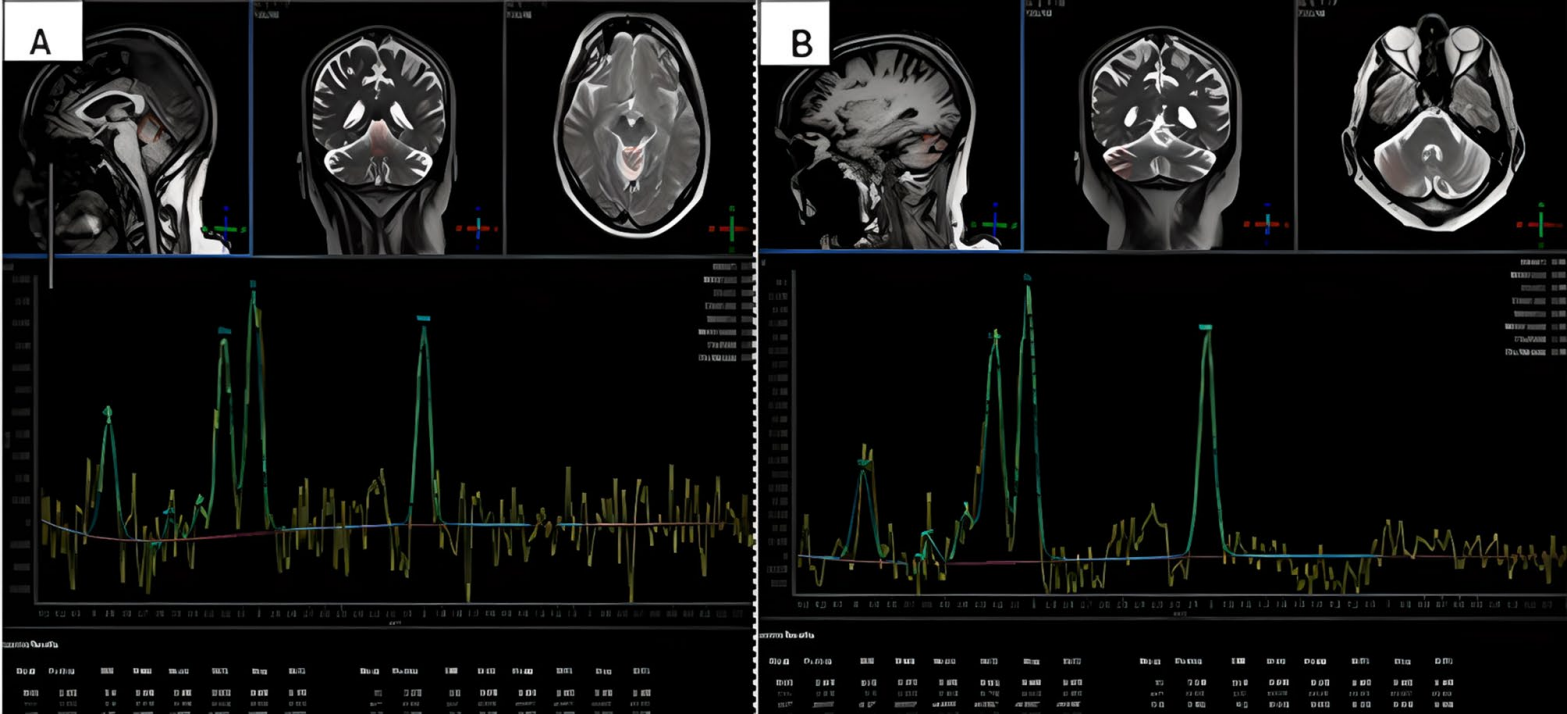
A representative single-voxel MR spectroscopy from the vermis (**A**) and right cerebellar hemisphere (**B**), demonstrating reduced NAA/Cr ratios consistent with impaired neuronal integrity

In 21 (42%) patients, longitudinal analysis of MR Spectroscopy (MRS) was obtained, over a mean duration of 44 months (excluding individuals on 3,4-DAP and/or incomplete MRS data), allowing assessment of disease progression. This showed the following: at baseline, the mean NAA/Cr ratio was 0.85 (SD ± 0.10) in the vermis and 0.96 (SD ± 0.16) in the hemisphere. In comparison, their most recent MRS measurements showed a mean NAA/Cr ratio of 0.87 (SD ± 0.16) in the vermis (such a change is within measurement error) and 0.90 (SD ± 0.14) in the hemisphere. Whilst the observed difference in the vermis was not statistically significant (*p* = 0.43,) the cerebellar hemisphere showed a statistically significant reduction over time (*p* = 0.033), suggesting progressive neuronal loss/dysfunction in the cerebellar hemispheres as depicted in Fig. 3.

**Fig. 4:**
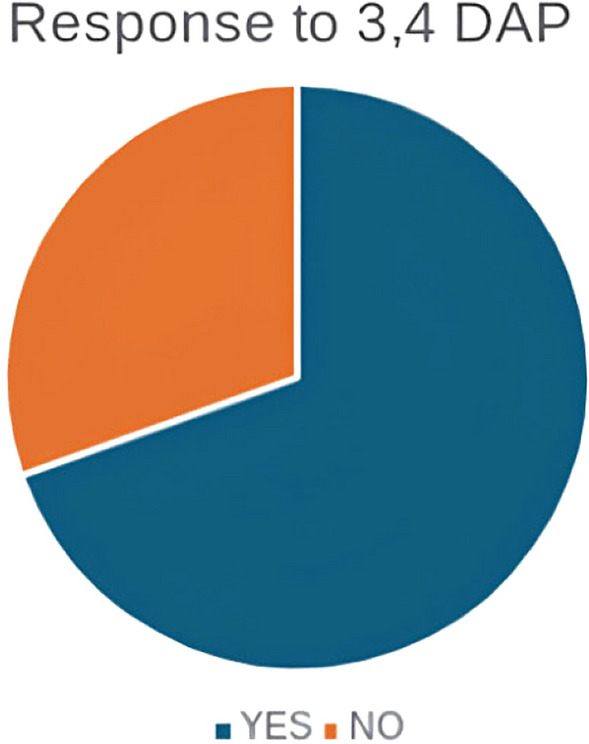
Response to 3,4-diaminopyridine in (*n* = 23) treated

Among this cohort, 23 patients were treated with 3,4-DAP, treatment was initiated at a starting dose of 10 mg three times daily (TDS) and if further dosing was required, and the initial dose was well tolerated, the dose was up-titrated to 20 mg TDS. A total of 70% of treated patients responded to 3,4-DAP, with most individuals stating overall improvement in vision, mobility, and coordination with a reduction in the severity and frequency of the ataxic episodes. There was no effect on the tremor. The remaining 27 (54%) patients are yet to try 3,4-DAP (see Fig. 4).

Within the cohort receiving treatment with 3,4-DAP, available MRS data from seven of the patients were analyzed to assess metabolic changes in the cerebellar vermis and hemispheres pre- and post-treatment. The mean NAA/Cr ratio in the vermis increased from 0.86 ± 0.07, before 3,4-DAP administration to 1.00 ± 0.05, following treatment. Similarly, the mean value in the hemispheres rose from 0.88 ± 0.08 to 0.98 ± 0.08. The analysis revealed a statistically significant increase in MRS values following treatment in both the vermis (median difference =  + 0.18, *W* = 0, *p* = 0.031) and the hemisphere (median difference =  + 0.11, *W* = 0, *p* = 0.031).

## Discussion

To our knowledge, this study represents the largest single-center cohort of patients with genetically confirmed SCA27B reported to date in the UK. Our results are consistent with earlier studies characterizing SCA27B as a slowly progressing, late-onset cerebellar ataxia [12].

In comparison to previous studies [1, 4, 9, 11], our patient cohort demonstrated a comparable mean age of onset (61.8), with a predominance of male patients and a positive family history observed in only 29.5% of cases.

Clinical presentation was typical with gait ataxia, which gradually worsened over time, with several other cardinal features of ataxia, including nystagmus, mostly downbeat, oscillopsia. Other reported visual symptoms included intermittent diplopia and blurred vision, probably related to the oscillopsia. Additionally, our patients also experienced dysarthria, and sensory and sensorimotor symptoms similar to what has been described in other cohorts [4, 13] Like in previous reports [14], out of the 10 cases who had an electrophysiological study, 70% demonstrated evidence of neuropathy revealing a sensorimotor length-dependent axonal neuropathy in 40%, while 30% showed findings consistent with sensory ganglionopathy. Additionally, a proportion (22%) of our patients had tremor, a feature that has also been reported in individuals with SCA27B [2]. Tremor analysis revealed an essential-like tremor of intermediate frequency but with an unusual resting component and significant lower limb involvement. Interestingly, the classic hallmarks of cerebellar tremor (high amplitude/low frequency, intention component) do not seem to be a feature in our cohort.

Episodic symptoms were reported in 28% of our patients, typically lasting from several minutes to hours, with the frequency differing amongst patients. This clinical feature is relatively uncommon amongst genetically determined spinocerebellar ataxias, other than episodic ataxias. This phenomenon is likely related to disruptions in ion channel function and excitatory neurotransmission, which may underlie the development of the symptoms in this subset of patients [15]

To our knowledge, this is the first report on SCA27B that provides data on magnetic resonance spectroscopy demonstrating primarily cerebellar vermis dysfunction, less so involvement of the cerebellar hemispheres. This is in keeping with gait ataxia being the presenting feature [3].

We have also demonstrated the involvement of the superior cerebellar peduncle, with prominent T2 MRI-weighted hyperintensity in this region, seen in 75.5% of patients. This has been reported in other studies [2, 16, 17], though these radiographic changes are not exclusive to this condition and may occasionally appear in other genetic ataxias [18]. This finding, however, may aid the diagnosis in the appropriate clinical context, as early symptoms of the disease can be subtle, particularly if episodic, and patients may lack clinical signs if assessed between attacks (including nystagmus).

Longitudinal MR spectroscopy was performed over a mean period of 44 months and showed a significant decline in the NAA/Cr area ratio in the cerebellar hemisphere but not the vermis for most patients. This is important and in keeping with the natural history of the condition, in that, despite the episodic nature the condition is progressive with accumulation of disability.

FGF14 modulates Purkinje cell firing through its effect on voltage-gated sodium channels and its regulation of voltage-gated calcium channels [19]. Studies using FGF14 knockdown mice have shown decreased calcium currents and impaired presynaptic Ca^2+^ influx, which results in decreased excitatory postsynaptic currents. This suggests that FGF14 is involved in both sodium and calcium channel function, so it is not surprising that medications that act on these channels may play a role in SCA27B symptomatology by [20] helping to reverse the decreased cell firing of Purkinje and granule cells after depolarization [21]. Aminopyridines enhance the resting activity and excitability of Purkinje cells in the cerebellum, potentially restoring their inhibitory control over the deep cerebellar nuclei. Aminopyridines have demonstrated significant efficacy in alleviating symptoms associated with cerebellar dysfunction, particularly DBN and oscillopsia. These improvements are primarily attributed to a reduction in the slow-phase velocity of DBN, alongside enhancements in gait stability [22]. This is in keeping with the therapeutic approaches that have been proposed, of the use of aminopyridine compounds in patients with SCA27B, along with the noted tolerability of these medications observed in other studies [7, 20].

Accessibility in the use of 4-aminopyridine is limited in the UK due to cost and the fact that its use in SCA27B is not yet a licensed indication. By contrast, 3,4-DAP is much cheaper in the UK and, as such, much more practical to use even if it is not yet a licensed treatment for this condition. We demonstrate here, for the first time, that patients with SCA27B receiving 3,4-DAP showed clinical (70% of patients improved) and MR spectroscopic improvements when compared to those who did not take the medication and the small proportion who stopped it due to intolerance. This is an important and novel finding as, to our knowledge, no other group has reported the beneficial effects of 3,4-DAP.

Although 4-aminopyridine is typically favored by some clinicians due to its lipophilicity, which facilitates passage across the blood–brain barrier (BBB), 3,4 DAP, being more hydrophilic, exhibits limited BBB penetration [23]. Our clinical experience, however, supports the therapeutic potential of 3,4-DAP in patients with SCA27B, demonstrating consistent radiological and clinical improvements. These findings point to a possible role of 3,4-DAP in enhancing neuronal function, particularly in the cerebellum. These preliminary results highlight the need for further studies through controlled clinical trials where a direct comparison between 4-aminopyridine and 3,4-DAP should be made. Furthermore, 3,4-DAP has shown favorable tolerability and a markedly better cost-effectiveness profile. These findings support the pragmatic use of 3,4-DAP as a viable alternative to 4-aminopyridine in this patient population.

SCA27B results from an expansion of GAA·TTC repeats of 250 or more in the first intron of the *FGF14* gene. Molecular genetic testing for SCA27B is currently performed using long-range PCR and repeat-primed PCR to determine repeat size and purity, with non–GAA-pure alleles lacking typical stutter patterns requiring further characterization by Sanger or long-read sequencing [9, 24, 25]. Current sequencing approaches, including multigene panels, exome sequencing, and short-read genome sequencing, are unable to reliably detect pathogenic FGF14 expansions, as their sensitivity is limited to repeats of approximately 50 triplets or fewer [2]. The exact threshold for what constitutes a pathogenic expansion is still being debated [26]. An expansion of 250 to 300 repeats may be considered pathogenic, though it exhibits reduced penetrance, while expansions exceeding 300 repeats are deemed highly penetrant [27]. There is a grey area regarding individuals with repeat expansions ranging between 200 and 250, and in our cohort, we identified eight such cases that were included in this series. Within the same family of three affected individuals who have the typical clinical features of SCA27B, two had GAA expansion sizes of 245 and 248, respectively, and the third had an expansion size of 259 repeats. In our cohort of patients as a whole, those with expansions of more than 250 and those with expansions between 200 and 250 demonstrated comparable demographic characteristics and clinical phenotypes. This observation is consistent with the findings of Pellerin et al. [6, 26]*.* Therefore, we believe that these two patients with expansions under 250 also have SCA27B.

The observed instability of GAA repeats during intergenerational transmission, specifically, expansion via maternal inheritance and contraction via paternal inheritance[26], adds further complexity to the interpretation of pathogenicity within this range. Gathering more clinical data and following up all patients with expansions under 250 may offer some eventual clarity for pathogenicity.

Notably, some individuals in our study also had a definitive diagnosis of alternative genetic ataxia, including one patient with the typical phenotype of cerebellar ataxia with neuropathy and vestibular areflexia syndrome (CANVAS) and another patient with SCA34 (both had expansions in the 250–300 range). One could argue that, given that SCA27B is proving very common, inevitably, there may be cases of dual pathology [6, 28]. The proportional clinical impact of each genetic defect is impossible to evaluate but may be of interest. One can argue that since SCA27B ataxia is a late-onset and mild ataxia that the phenotypes of the other genetic ataxias are likely to dominate the clinical picture [10, 28].

Thus, we advocate that patients with late-onset ataxia with or without family history and no alternative diagnosis should be tested for SCA27B. Improved diagnostic yield is likely by targeting those patients with episodic symptoms, prominent nystagmus (particularly downbeat), and with characteristic neuroimaging findings.

## Conclusion

SCA27B is an increasingly recognized cause of late-onset cerebellar ataxia often associated with DBN. While neuroimaging findings such as cerebellar atrophy and bilateral superior cerebellar peduncle T2 hyperintensities can support the diagnosis, a definitive diagnosis relies on the identification of a pathogenic expansion of the GAA repeat through genetic testing. Furthermore, our findings provide evidence for the potential efficacy of 3,4-DAP in improving symptoms in individuals with SCA27B and underscore the therapeutic promise of this medication, thus highlighting its use as an alternative to 4-aminopyridine.

## Data Availability

Not applicable.
